# Supplementary material for: Rapid and sensitive protein complex alignment with Foldseek-Multimer
Source: Nat Methods. 2025 Feb 5;22(3):469–72. doi: 10.1038/s41592-025-02593-7 (PMC11903335; doi:10.1038/s41592-025-02593-7)
Supplement: Supplementary file 1 — Supplementary Figs. 1–5 and Tables 1 (Supplementary Figs. 1–5 are also Extended Data) [file 41592_2025_2593_MOESM1_ESM.pdf]

nature methods

Supplementary information https://doi.org/10.1038/s41592-025-02593-7

Rapid and sensitive protein complex alignment with Foldseek-Multimer

In the format provided by the authors and unedited

Epsilon-delta	CV	Pearson's r	$\hat{b0}$	$\hat{b1}$	Avg. runtime (s)
0.1	0.1	0.97	-0.124	1.107	605
0.05	0.1	0.97	-0.124	1.107	601
0.01	0.1	0.97	-0.124	1.107	603
0.1	0.05	0.94	-0.119	1.101	600
0.1	0.01	0.85	-0.129	1.106	591
0.1	0.2	0.97	-0.124	1.107	600

Supplementary Table 1. Effect of parameters on Foldseek-Multimer sensitivity and runtime. Foldseek-Multimer uses two hard-coded parameter values: epsilon-delta = 0.1 and CV = 0.1 (Online Methods, "Algorithm"). Here, we tested the impact of alternative values for these parameters on Foldseek-Multimer's sensitivity and runtime. Sensitivity was measured by fitting a linear regression model between US-align's TM-scores (the independent variable X) and Foldseek-Multimer's TM-scores (the dependent variable Y) computed for the dataset of 931 structurally-similar complex pairs of Fig. 2a. We then examined the estimates for the coefficients of the model $Y = b0 + b1 \times X$ as well as the correlation between X and Y (expressed as Pearson's *r*) for each alternative parameter setting compared to the default (first row). Lower (but not higher) values of the DBSCAN parameter epsilon-delta have the potential of improving sensitivity, however we did not observe any improvement over the default in either setting, with similar runtimes (averaged over 5 runs). We tested two more conservative CV cutoffs for the superposition matrix column exclusion, which resulted in worse correlations between Foldseek-Multimer and US-align compared to the default. This suggests a lower CV cutoff introduces more noise and is therefore less preferable. We also tested a higher value of CV, which resulted in the same correlation as the default. Taken together, these results support the choice of the hard-coded parameter values.

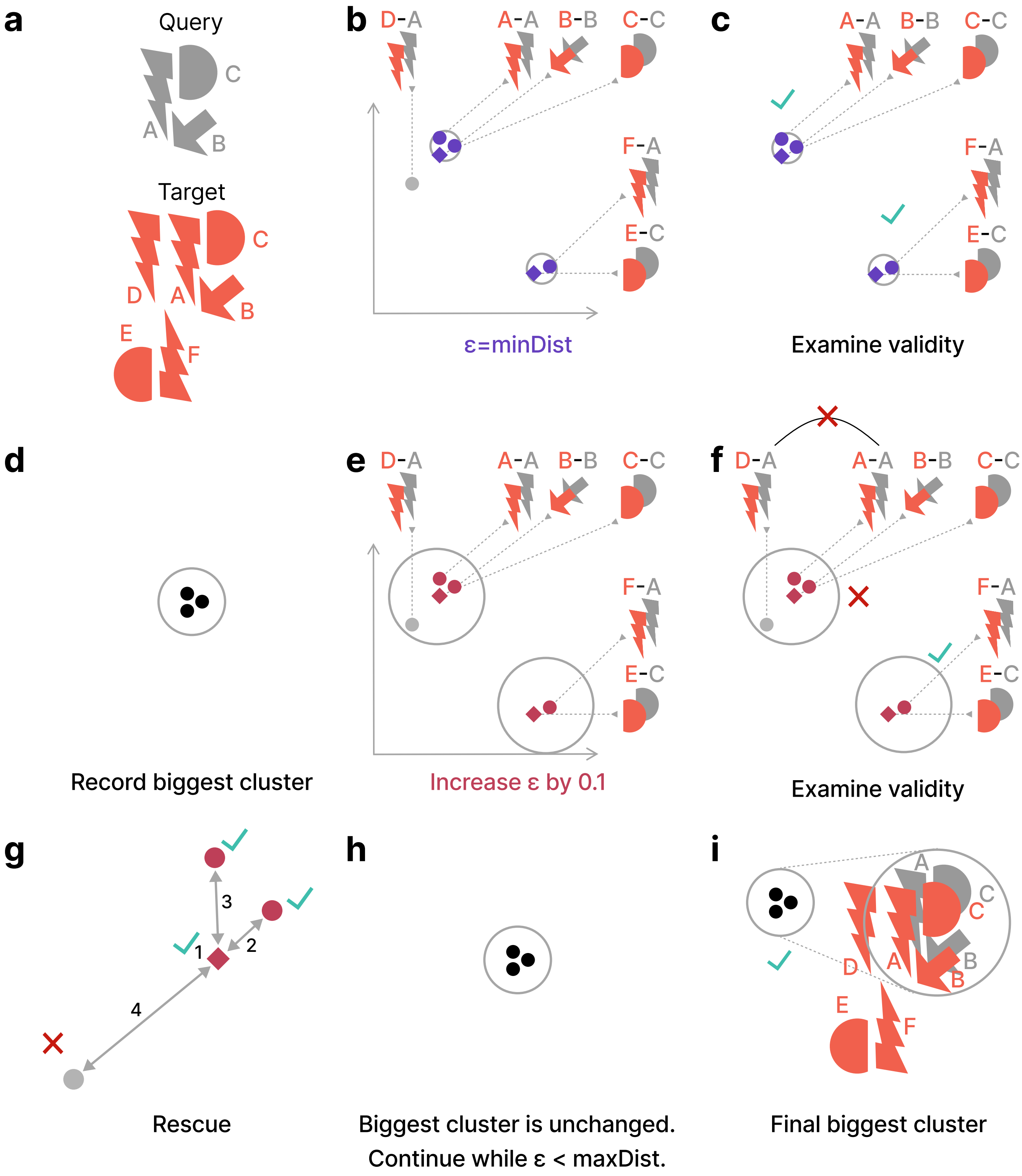

Supplementary Figure 1. Iterative DBSCAN procedure for clustering chain-to-chain alignments. The procedure steps are demonstrated schematically on a small example and fully described in the Online Methods section "Algorithm: Chain-to-chain clustering". **a**, A query complex can match a target complex in two ways. The first, in which chains Q(A,B,C) are matched to T(A,B,C) is the best match with a certain superposition. The second, in which Q(A,C) are matched to T(F,E) is a sub-optimal match with a different superposition. In addition, query chain A can match target chain D with a third superposition, which is somewhat close to the best superposition (but not identical). **b**, after the chain-to-chain superpositions (points) are computed they are clustered using DBSCAN with $\epsilon = minDist$, the minimal distance between any two points. Five points have enough neighbors to be considered as core-points (purple) and two clusters are discovered. The core-points from which the clusters started are marked as diamonds. **c**, Both clusters are valid as they contain at least two chains and no chain is repeated. **d**, The biggest (most chains) cluster is recorded. **e**, ϵ is increased by 0.1 and a new DBSCAN clustering begins with core-points (burgundy) and discovers two clusters. **f**, One of the clusters is not valid since query chain A is matched twice to chains A and D on the target. **g**, In a rescue procedure, the points of the cluster are examined by their distance (numbered arrows) to the starting core-point (diamond), collecting a valid subset of points and stopping when a violating point is reached. **h**, The biggest valid cluster is unchanged in this example. The procedure continues until ϵ is greater than the distance between the two furthest points (maxDist). **i**, The final biggest cluster(s) discovered over all iterations is used for defining the complex-to-complex alignment and computing its TM-score.

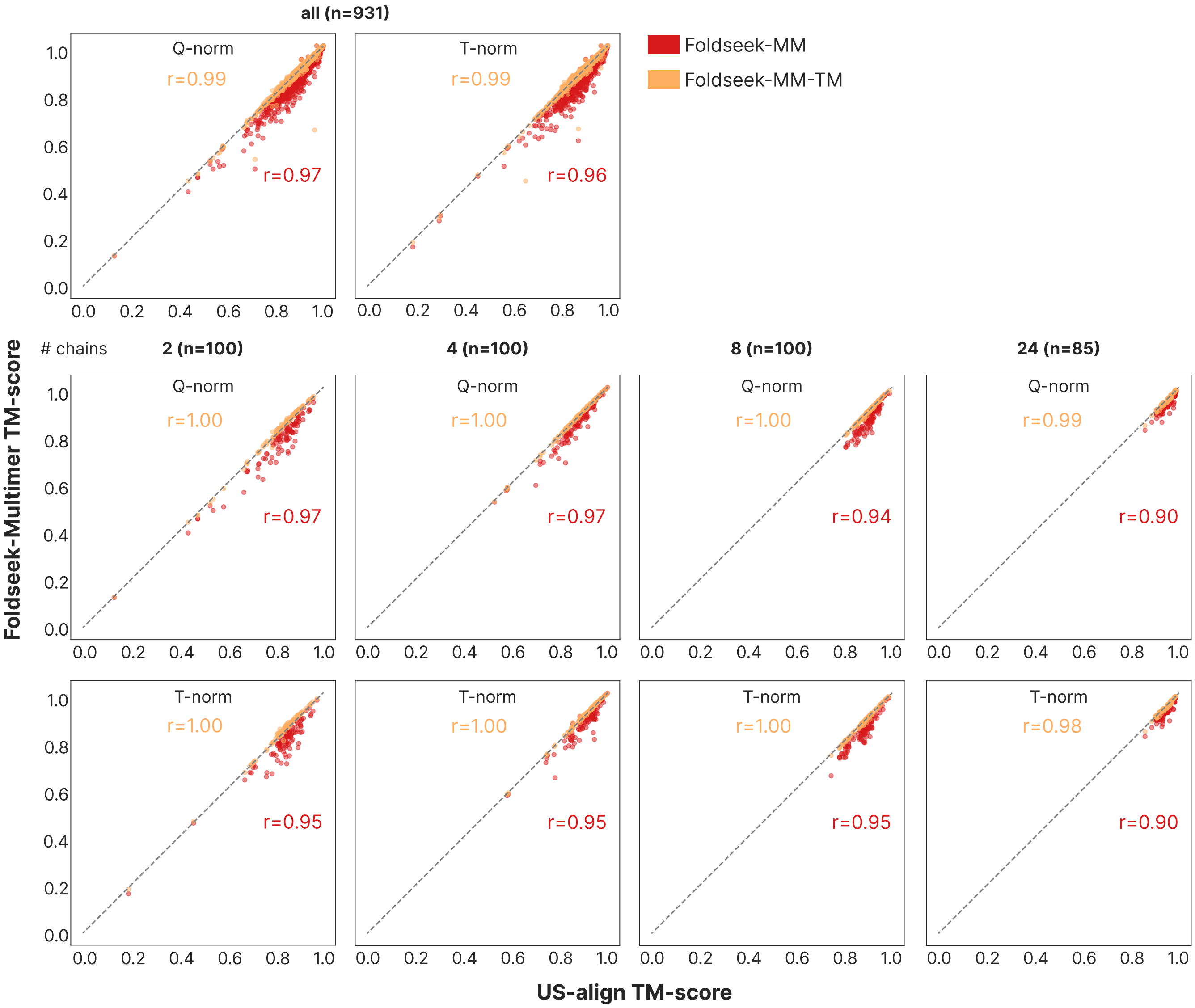

Supplementary Figure 2. TM-score correlation by normalization method and number of chains. Fig. 2a presents the correlation between query-length normalized (Q-norm) TM-scores computed by either Foldseek-Multimer or US-align for 931 pairs of structurally similar complexes. This computation was repeated using target-length normalization (T-norm), resulting in similarly very high correlation between the tools (top panel, right). In addition, the correlation was computed separately for complexes of the same number of chains using either query- or target-length normalization if there were at least 30 complexes of that size (i.e., separately for complexes with 2, 3, 4, 5, 6, 8, 10, 12, 14, and 24 chains). For all computed cases, the correlation was found to be high (Pearson's *r*: 0.90-1.0). The middle and bottom panels depict these correlations for a subset of the separately-computed cases. The sample size n is indicated in parentheses.

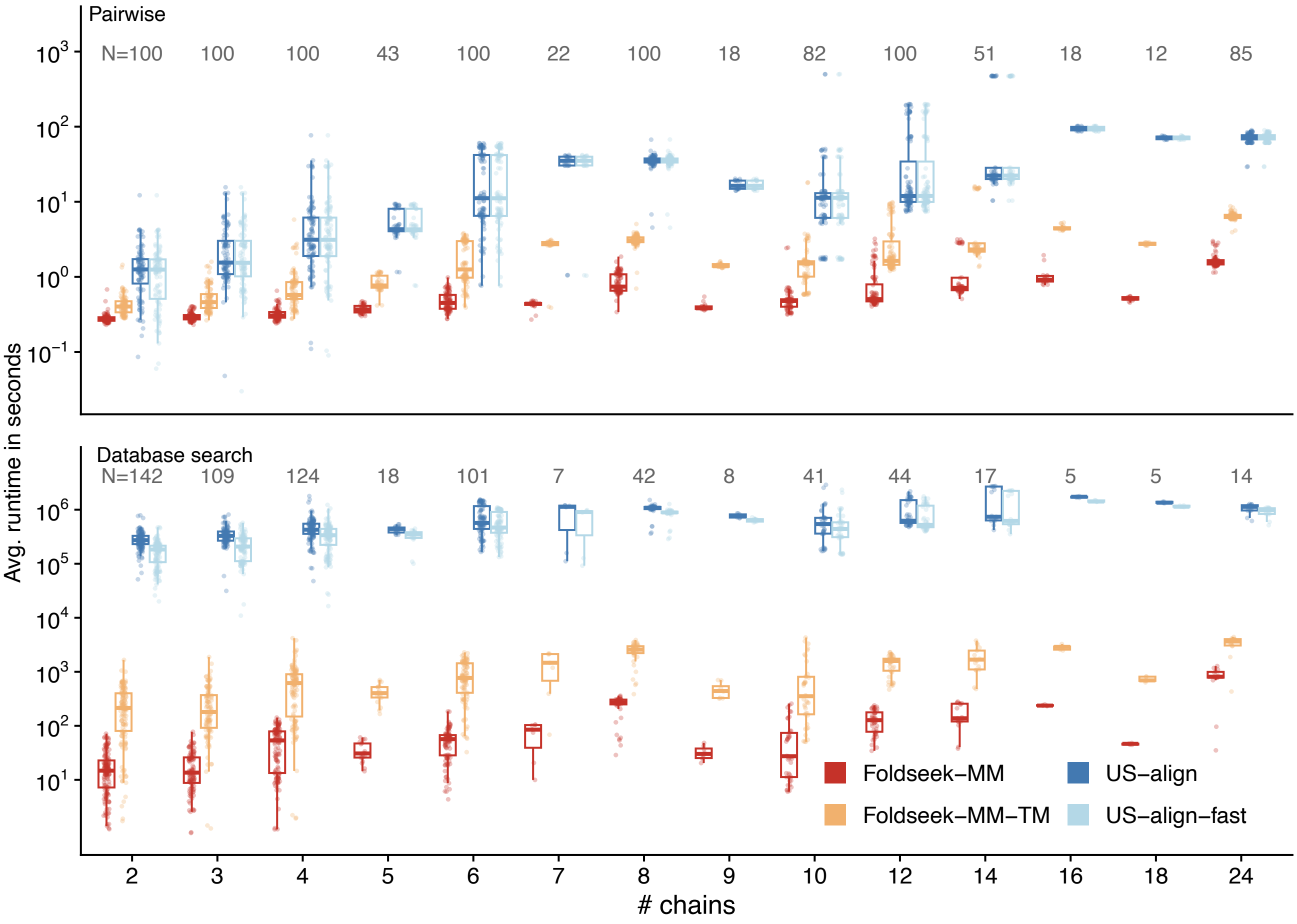

Supplementary Figure 3. Speed comparison of Foldseek-Multimer to US-align. Execution time based on the dataset used for Fig. 2a-b. Complexes were binned by their number of chains. Speed comparison of pairwise alignment (top) and database search (bottom). Boxplots depict quartiles, each point is a complex-pair (top) or complex (bottom), sample sizes indicated as *N*, whiskers drawn to maximum (minimum) point within 1.5 $\times$ the interquartile range over (under) 75th (25th) percentile. Pairwise mode (top): Foldseek-Multimer is $\times 10 - 100$ faster than US-align due to efficient chain-to-chain alignment and superposition clustering. Database search (bottom): complexes were queried against 3DComplexV7. Foldseek-Multimer is further accelerated by its prefilter, making it $\times 10^3$-10^4 faster.

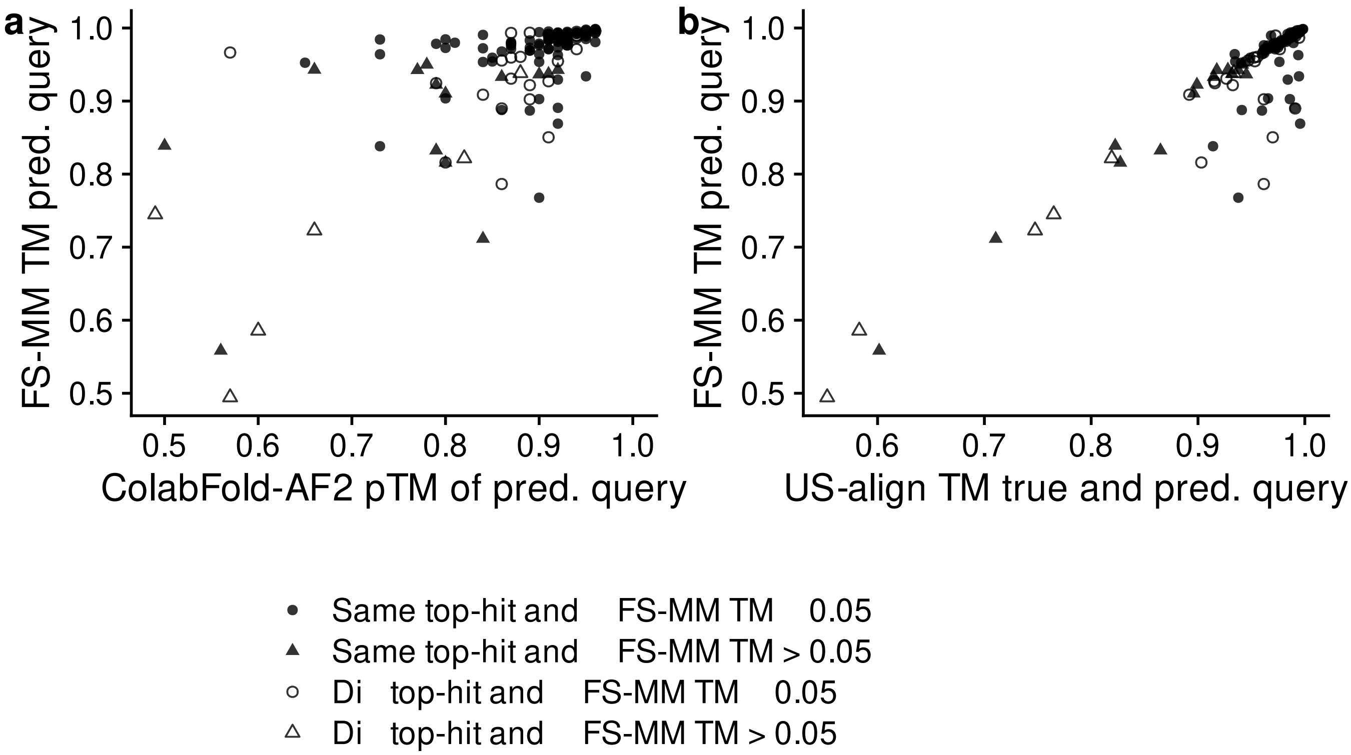

c

	Diff hit and Δ>0.05	Diff hit and $\Delta \leq 0.05$	Same hit and Δ>0.05	Same hit and $\Delta \leq 0.05$
pTM_high (>0.8)	2	27	5	75
pTM_moderate (0.5-0.8)	3	3	8	8

Supplementary Figure 4. Effect of complex structure prediction quality on Foldseek-Multimer. The results presented in Fig. 2a are based on PDB structures, which have been determined using gold-standard techniques and can be considered as "true". Here, we sought to measure the effect of using predicted query structures, as would be the case in metagenomic studies, like the analysis presented in Fig. 2c. To that end we used ColabFold-AlphaFold2 v1.5.5 to predict 50 (5 models ×10 seeds) structures for each of the 132 unique dimers, which were shorter than 1,000 residues, from the Fig. 2a dataset. We set the prediction parameters to produce structures of various qualities (`--use-dropout --num-seeds 10 --num-recycle 6`). For each of the dimers, we took the lowest ranked prediction. The quality of the predicted queries was measured in two ways: by the pTM score, estimated by ColabFold-AlphaFold2 and by the US-align TM-score between the true and predicted structures. While the latter is more accurate, this information will not be available in a metagenomic study, where the true structure is unknown. We then used Foldseek-Multimer to search each predicted query complex against the PDB100 database, recorded its top hit and score, and compared it to the top hit and score recorded for the true structure. **a and b**, higher quality predictions tend to result in better FS-MM TM-scores. At the same time, despite the variance in prediction quality, all but three (97.7%) predicted structures had a FS-MM top hit with a TM-score > 0.65. Of these, most (85.6%) had the a very small FS-MM TM-score difference ($\leq$0.05) when querying with the predicted and the true structure (circles). **c**, Predicted structures that were less similar to the true one (pTM_moderate) were more likely to have a worse score (triangles, Δ = FS-MM score true query - FS-MM score predicted query) compared to well predicted structures (pTM_high). A two-sided chi-square test of independence was performed to examine the relation between the quality of the predicted structure and the difference in score. This association is statistically significant (Chi-square statistic = 29.3, N = 131, DF = 1, P-value = $6.11 \text{x} 10^{-8}$).

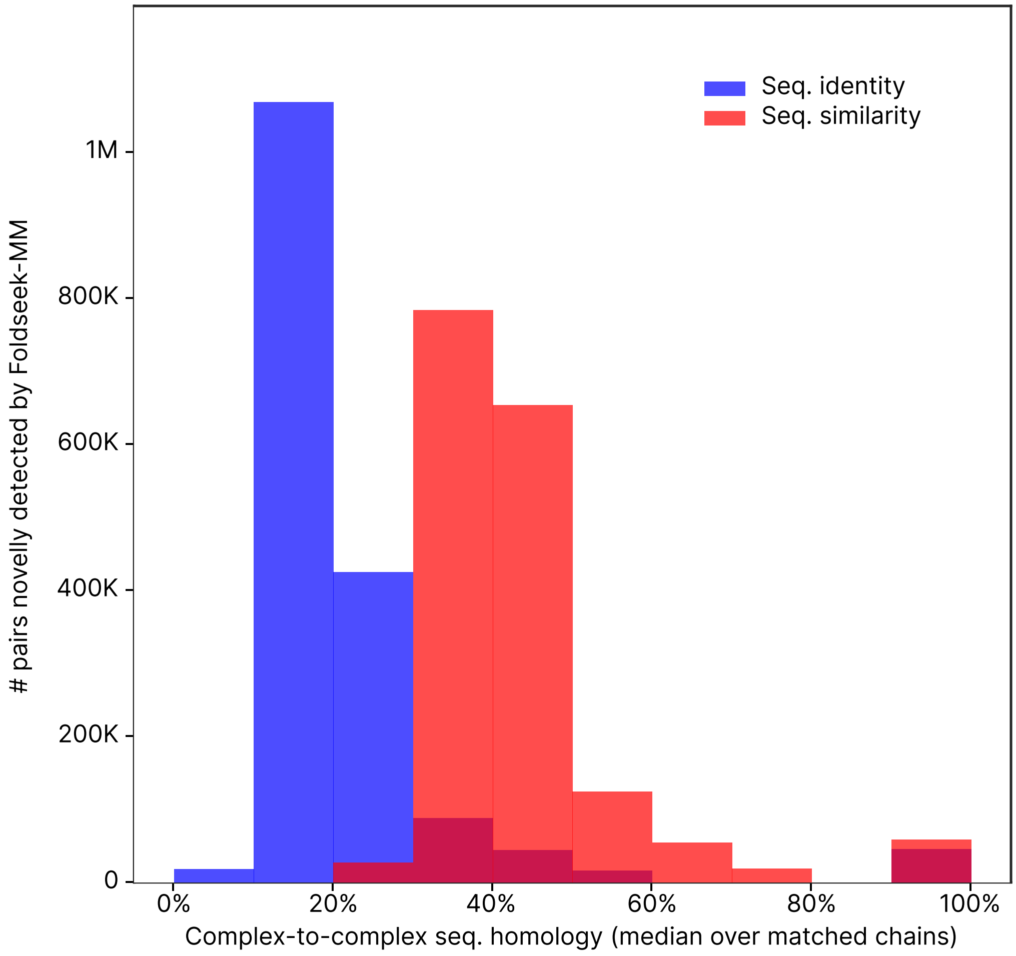

Supplementary Figure 5. Comparison of 3DComplexV7 complex pairs novelly discovered by Foldseek-MM. When applied to the 3DComplexV7 database, Foldseek-MM novelly reported 1,731,400 pairs of homomeric complexes as structurally similar (**Fig. 2d**). For these pairs we measured the median sequence identity (blue) or similarity (red, computed using the BLOSUM62 substitution matrix) over all matched chains of that pair. Most complex pairs (85%) were highly diverged, having less than 50% sequence similarity. Furthermore, these results demonstrate Foldseek-Multimer's ability to detect structural similarity well below the twilight zone as 87% of the pairs had less than 30% sequence identity between the complexes.
