# Supplementary material for: Rapid and sensitive protein complex alignment with Foldseek-Multimer
Source: Nat Methods. 2025 Feb 5;22(3):469–72. doi: 10.1038/s41592-025-02593-7 (PMC11903335; doi:10.1038/s41592-025-02593-7)
Supplement: Supplementary file 2 — Reporting Summary [file 41592_2025_2593_MOESM2_ESM.pdf]

nature portfolio

Corresponding author(s): Emmanuel D Levy, Martin Steinegger

Last updated by author(s): Oct 22, 2024

Reporting Summary

Nature Portfolio wishes to improve the reproducibility of the work that we publish. This form provides structure for consistency and transparency in reporting. For further information on Nature Portfolio policies, see our Editorial Policies and the Editorial Policy Checklist.

Statistics

For all statistical analyses, confirm that the following items are present in the figure legend, table legend, main text, or Methods section.

n/a	Confirmed	
☐	☒	The exact sample size (*n*) for each experimental group/condition, given as a discrete number and unit of measurement
☐	☒	A statement on whether measurements were taken from distinct samples or whether the same sample was measured repeatedly
☐	☒	The statistical test(s) used AND whether they are one- or two-sided<br>*Only common tests should be described solely by name; describe more complex techniques in the Methods section.*
☒	☐	A description of all covariates tested
☒	☐	A description of any assumptions or corrections, such as tests of normality and adjustment for multiple comparisons
☐	☒	A full description of the statistical parameters including central tendency (e.g. means) or other basic estimates (e.g. regression coefficient) AND variation (e.g. standard deviation) or associated estimates of uncertainty (e.g. confidence intervals)
☐	☒	For null hypothesis testing, the test statistic (e.g. *F*, *t*, *r*) with confidence intervals, effect sizes, degrees of freedom and *P* value noted<br>*Give P values as exact values whenever suitable.*
☒	☐	For Bayesian analysis, information on the choice of priors and Markov chain Monte Carlo settings
☒	☐	For hierarchical and complex designs, identification of the appropriate level for tests and full reporting of outcomes
☐	☒	Estimates of effect sizes (e.g. Cohen's *d*, Pearson's *r*), indicating how they were calculated

Our web collection on statistics for biologists contains articles on many of the points above.

Software and code

Policy information about availability of computer code

Data collection	Software used in benchmark :<br>- Foldseek(commit c27a629) https://github.com/steineggerlab/foldseek/<br>- USalign (version 20220924) https://zhanggroup.org/US-align/bin/module/USalign.cpp
Data analysis	The analysis scripts are publicly available at https://github.com/steineggerlab/foldseek-multimer-analysis.<br>The anaysis data are publicly available at https://doi.org/10.5281/zenodo.13121434.

For manuscripts utilizing custom algorithms or software that are central to the research but not yet described in published literature, software must be made available to editors and reviewers. We strongly encourage code deposition in a community repository (e.g. GitHub). See the Nature Portfolio guidelines for submitting code & software for further information.

Data

Policy information about availability of data

All manuscripts must include a data availability statement. This statement should provide the following information, where applicable:

- Accession codes, unique identifiers, or web links for publicly available datasets
- A description of any restrictions on data availability
- For clinical datasets or third party data, please ensure that the statement adheres to our policy

Databases:

- 3DComplex (version V7) https://doi.org/10.5281/zenodo.13121434
- PDB (November 2023) files.wwpdb.org/pub/pdb/data/structures/all

Human research participants

Policy information about studies involving human research participants and Sex and Gender in Research.

Reporting on sex and gender	n/a
Population characteristics	n/a
Recruitment	n/a
Ethics oversight	n/a

Note that full information on the approval of the study protocol must also be provided in the manuscript.

Field-specific reporting

Please select the one below that is the best fit for your research. If you are not sure, read the appropriate sections before making your selection.

☒ Life sciences ☐ Behavioural & social sciences ☐ Ecological, evolutionary & environmental sciences

For a reference copy of the document with all sections, see nature.com/documents/nr-reporting-summary-flat.pdf

Life sciences study design

All studies must disclose on these points even when the disclosure is negative.

Sample size

The 3DComplex database
We downloaded the 3DComplex database version 7 (3DComplexV7DB). This database holds 238,965 structures, consisting of 557,146 chains and was created from the “Biological Units/Assemblies” downloaded from the PDB (Levy et al.).

Pairwise benchmark
Starting with the list of 57,953,513 QSalign-compared 3DComplexV7 structures, pairs of complexes were selected per number of subunits, with that number ranging from 2 to 24. For each size, the criteria for selection were that the TMscore computed by Kpax was greater than 0.8, and that pairs of homomers had less than 80% sequence identity. If more than 100 pairs matched the criteria, only the first 100 were selected, resulting in a total of 931 complex pairs included in the benchmark. These sample sizes are sufficient because we either collected all pairs in the database that matched the similarity criteria (so no sampling) or sampled 100, which is sufficient according to the central limit theorem as we report the difference in average runtimes. We could not perform the measurement on all pairs if there were more than 100, because of USalign's prohibitively slow runtimes.

PDB100 database
First, PDB, containing the asymmetric unit of 207,937 entries, consisting of 1,047,615 chains, was downloaded in November 2023 (Data Availability). Of these, 11,901 entries were associated with more than one structural model (e.g., the NMR experiment 2KOX). In total, 426,347 structural models were associated with the PDB entries. Next, all chains were clustered using Foldseek resulting in 343,785 redundancy reduced representatives.

Data exclusions	We extrapolated runtime of US-algin from a random sample of 1000 structures in Fig2b(bottom) and Fig.2c otherwise no data was excluded.
Replication	We checked that our method produces deterministic results. We published scripts and data to make the study reproducible.
Randomization	No randomization was performed since it was not relevant to this study. Not a comparison across groups - each tool analyzes all data points.
Blinding	No blinding was performed as it is not relevant to this study. Not a comparison across groups - each tool analyzes all data points.

Reporting for specific materials, systems and methods

We require information from authors about some types of materials, experimental systems and methods used in many studies. Here, indicate whether each material, system or method listed is relevant to your study. If you are not sure if a list item applies to your research, read the appropriate section before selecting a response.

Materials & experimental systems

n/a	Involved in the study
☒	☐ Antibodies
☒	☐ Eukaryotic cell lines
☒	☐ Palaeontology and archaeology
☒	☐ Animals and other organisms
☒	☐ Clinical data
☒	☐ Dual use research of concern

Methods

n/a	Involved in the study
☒	☐ ChIP-seq
☒	☐ Flow cytometry
☒	☐ MRI-based neuroimaging
